# Classification of variant portal vein anatomy based on three-dimensional CT: surgical implications

**DOI:** 10.1007/s00276-024-03427-5

**Published:** 2024-07-04

**Authors:** Zheyu Liu, Tianni Shen, Kexin Xia, Junye He, Tianhao Rui, Wei Chen

**Affiliations:** 1 Department of Biliary-Pancreatic Surgery, Renji Hospital, Shanghai Jiao Tong University School of Medicine, 160 Pujian Road, Shanghai, 200127 P.R. China; 2 Shanghai Jiao Tong University School of Medicine, Shanghai, 200025 P.R. China; 3 Shanghai Jiao Tong University, Shanghai, 200240 P.R. China

**Keywords:** Anatomic variation, Clinical practice, Portal vein, Three-dimensional imaging

## Abstract

**Purposes:**

The purpose of this study was to develop a new and more comprehensive classification system for portal vein (PV) variations using three-dimensional visualization and evaluation (3DVE) and to discuss the prevalence rates and clinical implications of the variants.

**Methods:**

The anatomies of PVs were tracked and analyzed by using three-dimensional visualization of CT images acquired between 2013 and 2022. Scans from 200 adults were evaluated and a total of 178 patients (*N* = 178) were included in the study. The new classification system, named BLB classification, was developed based on the level of the absent PV branch in each variant anatomy.

**Results:**

Using the BLB classification system, PVs were divided into thirteen subtypes. Only 82.6–84.8% of the portal veins of the 178 patients were depicted in Atri’s, Cheng’s or Covey’s classification, compared with 100% identified by the BLB classification. The BLB classification was validated against external data sets from previous studies, with 97.0-98.9% of patients classified by the BLB system.

**Conclusion:**

Variant PV anatomies are more commonly seen based on 3DVE than in previous reports. The BLB classification covers almost all portal vein variants and may be used for planning liver surgery.

## Summary Box

- **What is already known on this topic**: To date, the classification defined by Atri et al. and Covey et al. are mostly used, including four and six subtypes respectively. However, variants and patterns not defined in traditional classifications are frequently encountered during surgery.
- **What this study adds**: In this article, we have explored the development of a new classification system (BLB classification) for portal vein anatomies based on three-dimensional visualization and evaluation. The importance of the finding is that the BLB classification covers almost all portal vein variants, which should be much more comprehensive than other reported classification methods and may be used for planning liver surgery.
- **How this study might affect research, practice or policy**: The knowledge of variant portal vein subtypes is critically significant in performing safe and efficacious liver surgeries, such as portal vein embolization, liver resection, liver transplantation and TIPS creation.

## Introduction

The portal vein (PV) is the main blood vessel that delivers blood to the liver from the gastrointestinal tract, including the stomach, intestines, spleen, and pancreas. The liver has a dual blood supply derived from the PV and the hepatic artery, most of which comes from the PV. A thorough knowledge of the course and variants of the PV is essential for surgical planning to prevent postoperative complications after liver surgery. In a liver transplantation surgery, for example, evaluation of the PV is of great surgical importance to the anastomosis of the recipient portal vein, as well as the repair of the donor’s residual liver function.

Atri et al. [1] defined a PV classification in which six types were listed. Afterwards Covey et al. [2] was alive to the prevalence and importance of third-level branch variants and brought forward two new subtypes. However, variants and patterns that were not defined in traditional classifications, such as those of Atri et al. [1] and Covey et al. [2], are frequently encountered during surgery. A classification of portal veins that covers almost all the anatomical variants would therefore be useful.

Traditional classifications of PVs were established based on autopsy or two-dimensional CT (2DCT). Portal veins, especially those in rare and complex patterns, can be easily neglected or mistaken by the reader on the venous phase of 2DCT. By restoring the three-dimensional (3D) structure of the PV and the liver contour, the 3D visualization technique (3DVT) can track the branches and course of the main portal vein (MPV), left portal vein (LPV), right portal vein (RPV), right anterior portal vein (RAPV) and right posterior portal vein (RPPV) based on CT data [3–5]. The present study explored the development of a new system (branch-level based classification) based on 3D visualization and evaluation (3DVE).

## Methods

This study was approved by the Ethical Committee of Renji Hospital, School of Medicine, Shanghai Jiao Tong University (registration number: Renji18076). Before surgery, all patients were given details of treatment, including the procedure, risks and complications of 3D CT reconstruction and evaluation. All patients signed consent forms before surgery, including acceptance of an evaluation using a 3D reconstruction technique.

### Patients

We retrospectively reviewed 200 multidetector CT (MDCT) examinations performed in our institute between September 2013 and December 2022. We excluded patients who had a history of major upper abdominal surgery such as previous liver resections, as well as patients with portal vein thrombosis and insufficient filling of vessels due to large central tumors and other pathologies obscuring the assessment of PV variations. So finally, a total of 178 (*N* = 178) patients were included in the study. Our study group comprised 80 (44.9%) females and 98 (55.1%) male patients. Mean age of the patients was 55 ± 17 years (mean ± standard deviation).

### CT and data collection

All patients in this study underwent triphasic CT abdomen on a 64-slice CT scanner (LightSpeed™ VCT; GE Healthcare, Milwaukee, Wisconsin, USA) with a slice thickness for axial images of 1.25 mm, reconstruction slice thickness of 1.25 mm and a reconstruction interval of 1.25 mm. Non-ionic iodinated contrast (Iopamidol^®^ 370 mg/ml; Shanghai Bracco Sine Pharmaceutical Company, Shanghai, China) was injected at a rate of 3–4 ml/s. Hepatic arterial, portal venous and hepatic venous phase scans were acquired in 20–25 and 60–65 s respectively. Saved in DICOM format, the enhanced CT data were then transferred to an IQQA^®^-LIVER workstation (EDDA Technology, Princeton, New Jersey, USA) and processed into a 3D visualization model.

### Three-dimensional visualization

3D visualization processing was structured as a five-step procedure. Firstly, the original imaging data acquired from MDCT were processed into a 3D model. Secondly, the contour of liver, automatically reconstructed through region-growing method, was then modified manually. Thirdly, the PVs and hepatic veins were extracted from the portal venous phase while the hepatic arteries were extracted from the arterial phase. The images from both phases were subsequently registered and coalesced. Finally, along the course of the PVs and hepatic veins, the 3D transection was determined based on Couinaud liver segmentation [6, 7].

### BLB classification system

The branch-level based (BLB) classification system was formulated based on the level of the absent PV branch in each variant portal vein anatomy.

### Statistical analysis

Categorical variables are reported as numbers with percentages. Differences between groups were analyzed using the χ^2^ test. *P* < 0.050 was considered statistically significant. Statistical analysis was carried out using SPSS^®^ version 26.0 (IBM, Armonk, New York, USA).

## Results

Scans from 200 adult patients were reviewed and a total of 178 patients was included in this study. The 178 patients had no liver space-occupying lesion or portal vein thrombosis and possessed high-quality CT imaging in which third-level branches can be distinctly observed.

### Types of portal vein branching pattern in BLB classification system

**Fig. 1:**
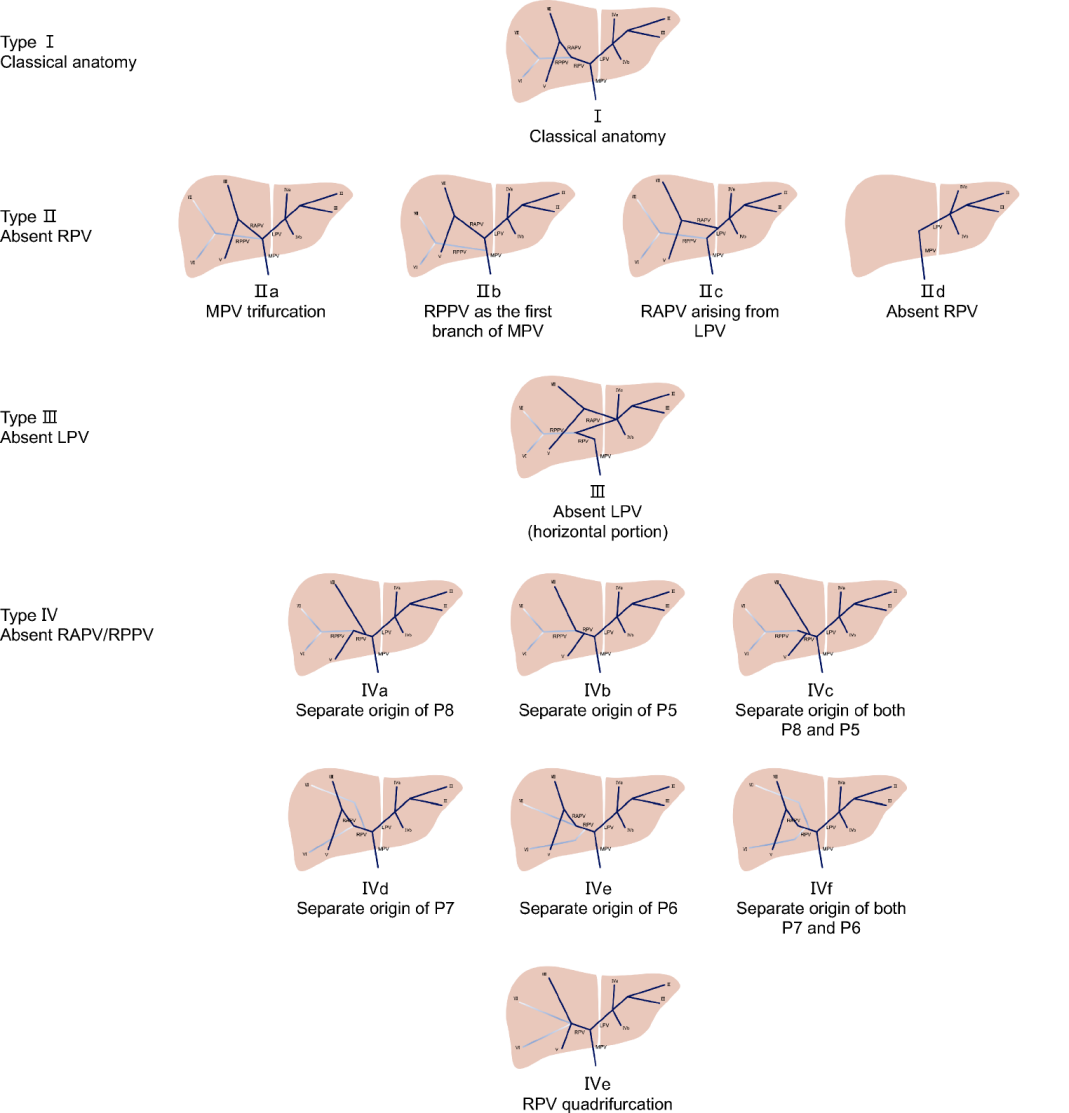
Schematic diagram of BLB classification system (Type I-Type IV). MPV, main portal vein; LPV, left portal vein; RPV, right portal vein; RAPV, right anterior portal vein; RPPV, right posterior portal vein; P8, segmental VIII branch; P5, segmental V branch; P7, segmental VII branch; P6, segmental VI branch

Based on preoperative 3DVE, 13 types of portal vein vasculature were described in 178 patients (Table 1; Fig. 1). Seven types of variants (28 patients, 15.7%) were not defined in Atri’s classification (Table 2). To simplify the BLB classification system and make it more practical, the 13 types of portal vein were summarized into four major categories.

**Table 1:** Types in BLB classification system based on portal veins of 178 patients

Classification	Description
Type I	*Classical anatomy*
Type II	*Absent RPV*
IIa	MPV trifurcation
IIb	RPPV as the first branch of MPV
IIc	RAPV arising from LPV
IId	Absent RPV
Type III	*Absent LPV (horizontal portion)*
Type IV	*Absent RAPV/RAPV*
IVa	Separate origin of P8
IVb	Separate origin of P5
IVc	Separate origin of both P8 and P5
IVd	Separate origin of P7
IVe	Separate origin of P6
IVf	Separate origin of both P7 and P6
IVg	RPV quadrifurcation

MPV, main portal vein; LPV, left portal vein; RPV, right portal vein; RAPV, right anterior portal vein; RPPV, right posterior portal vein; P8, segmental VIII branch; P5, segmental V branch; P7, segmental VII branch; P6, segmental VI branch

**Table 2:** Categorization of portal veins of 178 patients based on different classifications

BLB classification	Present cohort	Atri et al.(1)	Cheng et al.(6)	Covey et al.(2)
Type I	112 (62.9)	Normal Type: 112 (62.9)	Type 1: 112 (62.9)	Type 1: 112 (62.9)
Type II	37 (20.8)	37 (20.8)	35 (19.7)	30 (16.9)
IIa	23 (12.9)	Type 1a: 23 (12.9)	Type 2: 23 (12.9)	Type 2: 23 (12.9)
IIb	7 (3.9)	Type 1b: 7 (3.9)	Type 3: 7 (3.9)	Type 3: 7 (3.9)
IIc	5 (2.8)	Type 1c: 5 (2.8)	Type 4: 5 (2.8)	n.d.
IId	2 (1.1)	Type 1d: 2 (1.1)	n.d.	n.d.
Type III	1 (0.6)	Type 2: 1 (0.6)	n.d.	n.d.
Type IV	28 (15.7)	n.d.	n.d.	9 (5.1)
IVa	1 (0.6)	n.d.	n.d.	n.d.
IVb	1 (0.6)	n.d.	n.d.	n.d.
IVc	3 (1.7)	n.d.	n.d.	n.d.
IVd	6 (3.4)	n.d.	n.d.	Type 4: 6 (3.4)
IVe	3 (1.7)	n.d.	n.d.	Type 5: 3 (1.7)
IVf	6 (3.4)	n.d.	n.d.	n.d.
IVg	8 (4.5)	n.d.	n.d.	n.d.
Matching ratio*	178 of 178 (100)	150 of 178 (84.3)†	147 of 178 (82.6)†	151 of 178 (84.8)†

Values in parentheses are percentages. *Proportion of portal veins from 178 patients that could be depicted by each classification system. n.d., Portal vein configuration not defined in corresponding classification. †*P* < 0.001 versus matching ratio for BLB classification (χ^2^ test)

#### Type I

Type I is the normal anatomy; the MPV divides into the LPV and RPV, the RPV then bifurcates into the RAPV and RPPV which further subdivides into to two segmental branches supplying Couinaud liver segments VI and V and segments VII and VI, respectively.

#### Type II

Absent RPV; this is subdivided into four groups:

- Type IIa, MPV trifurcation; the MPV divides into three branches: the RAPV, the RPPV, and the LPV.
- Type IIb, the RPPV arises from the lower position of the MPV, and the MPV subsequently divides into a left and right anterior branch, scilicet the LPV and RAPV.
- Type IIc, the RAPV arises from the left portal branch.
- Type IId, complete absence of the RPV; this variant was considered to be associated with an absent or hypoplastic right lobe of liver [1].

#### Type III

Absent LPV; the horizontal portion of the LPV is absent, and the blood supply to the left lobe of liver comes through RAPV.

#### Type IV

Absent RAPV/RPPV; this is subdivided into seven groups:

Absent RAPV include separate origin of P8 (segmental VIII branch) from the RPV or right anterior portal veins (Type IVa), separate origin of P5 (segmental V branch) from the RPV or right anterior portal veins (Type IVb), and separate origin of both P8 and P5 from the RPV or right anterior portal veins (Type IVc). Absent RPPV include separate origin of P7 (segmental VII branch) from the RPV or right posterior portal veins (Type IVd), separate origin of P6 (segmental VI branch) from the RPV or right posterior portal veins (Type IVe), and separate origin of both P7 and P6 from the RPV or right posterior portal veins (Type IVf).

Type IVg, quadrifurcation of the RPV; absence of both the RAPV and RPPV, the RPV directly divides into P8, P5, P7 and P6.

### Alternative approaches to portal vein evaluation of 178 patients

Normal anatomy (Type I) was seen in 112 patients (62.9%) out of 178 patients in our study. MPV trifurcation variation (Type IIa) was seen in 37 (20.8%) of the cases. RPPV as the first branch of MPV variation (Type IIb) was seen in 23 (12.9%) of the cases. RAPV arising from LPV variation (Type IIc) was seen in 5 (2.8%) of the cases. Absence of RPV variation (Type IId) was seen in 2 (1.1%) of the cases. Absence of LPV variation (Type III) was seen in 1 (0.6%) of the cases. Absence of RAPV/RPPV variation (Type IV) was seen in 28 (15.7%) of the cases.

**Fig. 2:**
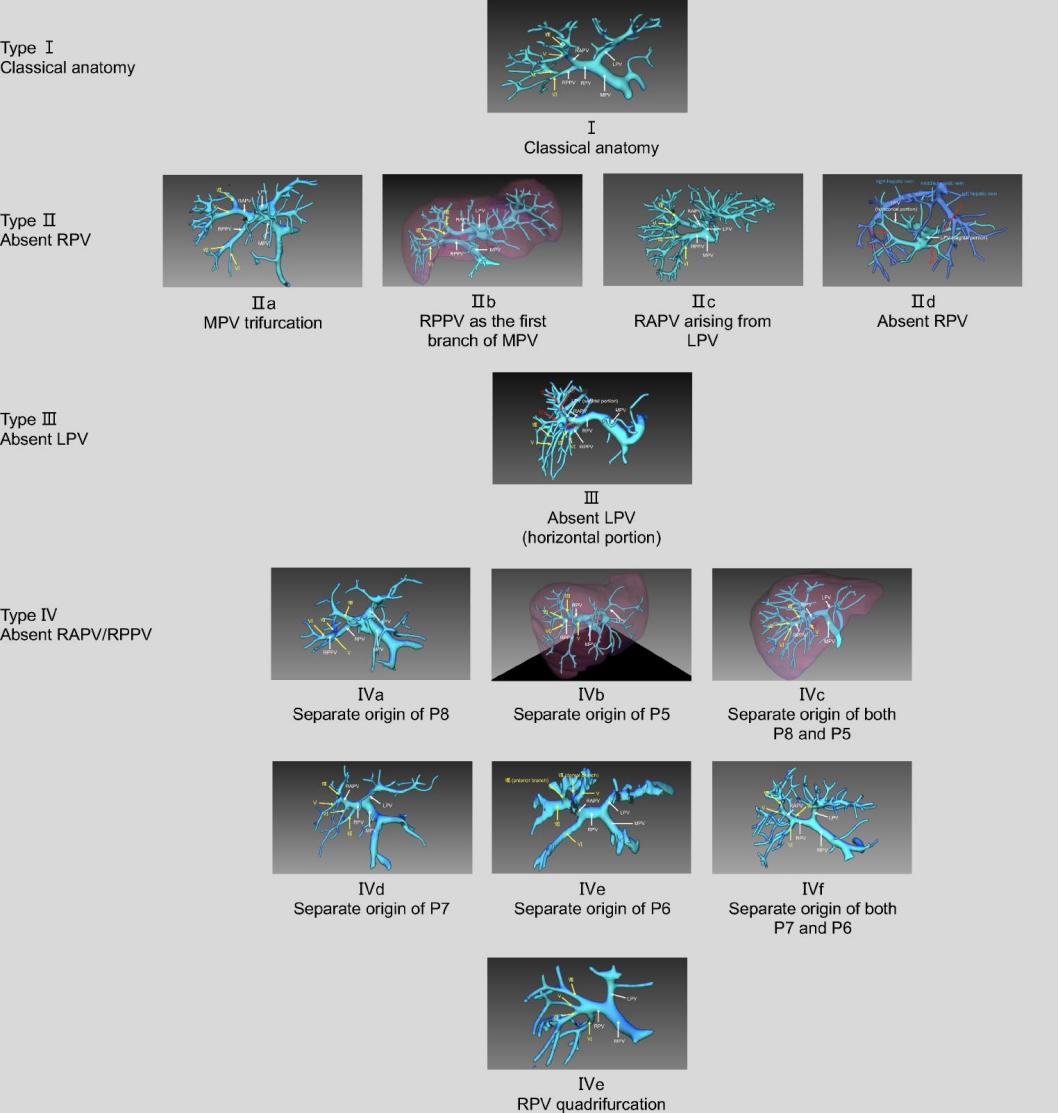
IQQA three-dimensional images showing all Type I-Type IV anatomies in BLB classification system. LPV, left portal vein; RPV, right portal vein; RAPV, right anterior portal vein; RPPV, right posterior portal vein; P8, segmental VIII branch; P5, segmental V branch; P7, segmental VII branch; P6, segmental VI branch

Different classifications of portal veins were compared based on 3DVE of 178 LDLT donors. The classification of Atri et al. [1], Cheng et al. [8] and Covey et al. [2] covered 150 (84.3%), 147 (82.6%) and 151 (84.8%) of the 178 patients respectively, whereas 100% could be defined and described using the BLB classification system (*P* < 0.001) (Table 2; Fig. 2). In particular, 35 and 30 of 37 portal vein configurations in Type II and 0 and 0 of 1 in Type III, according to BLB classification, could be described in Cheng’s and Covey’s classifications; and only 0, 0 and 9 of 28 in Type IV variation could be described in Atri’s, Cheng’s and Covey’s classifications. Specifically, Type IIc could not be defined in Covey’s classification, Type IId and III could not be defined in Cheng’s and Covey’s classification and were easily neglected. In addition, Type IVa (separate origin of P8), IVb (separate origin of P5), IVc (separate origin of both P8 and P5), IVf (separate origin of both P7 and P6), IVg (RPV quadrifurcation) could not be defined in all Atri’s, Cheng’s and Covey’s classifications.

### Further validation of BLB classification system

The BLB classification system was further validated using portal vein data extracted from four external data sets. The portal vein configurations could be depicted by Atri’s classification in 91.7% (887 of 967), 96.3% (1333 of 1384), 83.0% (83 of 100) and 87.0% (174 of 200), compared with 97.1, 98.9, 97.0 and 98.5% respectively by the BLB classification (*P* < 0.001) (Table 3).

**Table 3:** Portal vein types recorded in other studies re-evaluated based on BLB classification system and Atri’s classification

BLB classification	Sureka et al.(4) (*n* = 967)	Koç et al.(10) (*n* = 1384)	Gunasekaran et al.(9) (*n* = 100)	Covey et al.(2) (*n* = 200)
Type I	773 (79.9)	1045 (75.5)	67 (67.0)	130 (65.0)
Type II				
IIa	66 (6.8)	154 (11.1)	10 (10.0)	18 (9.0)
IIb	48 (5.0)	134 (9.7)	6 (6.0)	26 (13.0)
IIc				
IId				
Type III				
Type IV				
IVa	2 (0.2)			
IVb	5 (0.5)			
IVc				1 (0.5)
IVd	26 (2.7)	7 (0.5)	1 (1.0)	2 (1.0)
IVe	16 (1.7)	26 (1.9)	8 (8.0)	12 (6.0)
IVf	3 (0.3)	2 (0.1)		7 (3.5)
IVg		1 (0.1)	5 (5.0)	1 (0.5)
Matching ratio in BLB classification	939 of 967 (97.1)*	1369 of 1384 (98.9)*	97 of 100 (97.0)*	197 of 200 (98.5)*
Matching ratio in Atri’s classification	887 of 967 (91.7)	1333 of 1384 (96.3)	83 of 100 (83.0)	174 of 200 (87.0)

Values in parentheses are percentages. *P < 0.001 versus matching ratio in Atri’s classification for each study (χ^2^ test)

## Discussion

Embryologically, the portal vein is formed in the second month of gestation by selective involution of the vitelline veins, which have multiple bridging anastomoses anterior and posterior to the duodenum. Alterations in the pattern of obliteration of these anastomoses result in the anatomic variations of portal vein [2, 9–12].

In standard portal vein anatomy, the MPV, which carries as much as 80% of the blood supply to the liver, is formed by the union of splenic and superior mesenteric veins. The MPV subsequently divides into the LPV and RPV at the hilum. The RPV then bifurcates into the RAPV and RPPV, and the former divides into segment VI and segment V branches while the latter divides into segment VII and segment VI branches. The LPV initially has a horizontal portion to the left, which then courses medially towards the ligamentum teres giving branches to supply segments II, III and IV and the caudate lobe.

With the increase in percutaneous hepatobiliary interventions and complex surgical resections, a thorough understanding of standard and variant portal venous anatomy is critical. Previous studies have shown the prevalence of variant portal venous anatomy ranges from 20.1 to 35% [2, 5, 13, 14]. The prevalence in our series (37.1%) was slightly higher. In this study, we assessed portal vein anatomy by using 3DVT based on CT data to determine the patterns and incidence of variants in 178 patients.

The BLB classification system developed in the present study describes 13 subtypes of port vein, providing detailed information on the configurations of portal veins. To date, the classification defined by Atri et al. [1] in 1992 has been the most frequently used (6 types found in the autopsies of 507 patients). In 1996, Cheng et al. [8] simplified Atri’s classification into four types with minor revisions. In 2004, Covey et al. [2] reviewed the surgical records of 200 patients and proposed two new types of portal vein configurations, which are separate origins of segmental VII branch and segmental VI branch from the RPV. As a matter of fact, having been found in 33.5% of all cases in a cadaveric dissection study [15], variations in RPV ramification are not rare. Such variant of a single posterior segment branch may easily be overlooked but can have significant clinical consequences. And therefore, focus on the third-level branch variants has become prevailing in recent years [5, 13, 14].

In the present study, we found variant RPV ramification (Type IV) in 28 (15.7%) of 178 patients, which is consistent with the result of Atasoy et al. [16], who found 22 (16.8%) of 131 patients (*P* = 0.802). However, more emphasis has been laid on segmental VII branch and segmental VI branch variations (Type IVd-f) [2, 5, 14] when it comes to discussion on third-level branch variants. To the best of our knowledge, absence of RAPV (Type IVa-c) has not been previously described as a subtype of portal vein configurations in the literature. We found absence of RAPV (Type IVa-c) in 5 (2.8%) of 178 patients, which was significantly distinct from the results of Sureka et al. (7 of 967 patients, 0.7%) (*P* < 0.050) [5] and Koç et al. (0 of 1384 patients, 0.0%) (*P* < 0.001) [14]. This striking difference may have resulted from using different methods when imaging the portal venous configuration, because reformations in 3D are crucial for accurate visualization of the anatomy in such cases. When using 2D-images, separate origins of segment VII and VI branches from the RPV (Type IVd-f) can be easily mistaken for the sole cause of RPV trifurcation [16], which was considered as the most common variant of RPV ramification [15]. On the other hand, results based on 3DVE in the present study has revealed that RPV trifurcation may also be attributed to separate origins of segment VI and V branches (Type IVa-c). Knowledge of the third-level branch variants is crucial as for it may be beneficial in right anterior or posterior segment harvesting, as well as in segmental resection of the right lobe.

From the view of clinical practice, preoperative awareness of portal venous configuration is important before portal vein embolization, hepatic segments resection, graft procurement in liver transplantation and placement of transjugular intrahepatic portosystemic shunts (TIPS).

Transhepatic portal vein embolization is gaining acceptance as a method to induce contralateral liver hypertrophy in patients with small future remnant livers [17]. An understanding of variant portal vein anatomy is the prerequisite for safely and effectively performing embolization. Few technical difficulties are encountered when the anatomy of PV is normal. However, complexity arises in the case of Type IIb variation of PV, when a contralateral approach must be used and a reversed curved catheter may be required. In addition, variations like PV trifurcation (Type IIa) which can lead to difficult and unstable catheterization carry a higher risk for migration of embolic materials and thus result in non-target embolization [17–19]. And moreover, embolizing a non-targeted segment can make potentially resectable anatomy unresectable because of insufficient hypertrophy [2].

For a safe and clean hepatectomy, complete obliteration of the PV branches supplying those particular segments is required, which demands detailed evaluation of the PV branching pattern [5]. In the right hemihepatectomy for a Type IIb portal vein variant, the RPPV may easily be mistaken for the RPV and inadvertently ligated, leading to incomplete severance of portal venous flow of the right liver. In the case of left hemihepatectomy for a type IIc portal vein variant, the common trunk of the RAPV and LPV may easily be mistaken for the left branch and inadvertently ligated, leading to injury of the right anterior portal branch; and subsequently ischemic damage to hepatic segments VI and V. When performing segmental resection of the right lobe for a Type IV variant, resection of a particular lobe together with its third-level PV branch may devascularize a particular segment. For example, resection of segments VII and VI for a patient with separate origin of segmental VI branch from the RAPV (Type IVa) can result in devascularization of segment VI, leading to hypofunction of the remnant liver.

Anatomic variations of significance in liver transplantation surgery are Type IIa and Type IIb variations [20]. Trifurcation (Type IIa) variant increase the complexity as intraoperative clamping becomes difficult. Type IIb variant has its own surgical importance in recipient as well as in donor. In recipient, two PV anastomoses have to be performed, which is followed by the clamping involving both the RAPV and RPPV. In donor, the focus is on complete vascularization of remnant liver [21]. Presurgical evaluation of PV branching type should also pay attention to third-level branch variants (Type IV), particularly for segment harvesting.

TIPS creation remains a challenging procedure because it involves the successful passage of a needle from a point of origin (hepatic vein) to a target point (portal vein) through the liver substance [22]. A comprehensive knowledge of PV anatomy is critical for successful TIPS creations. In standard anatomy, the portal vein lies in a predictable position relative to the hepatic vein, accounting for high success rates [2]. However, in Type IIa and Type IIb variations, the puncture site of PV created during a TIPS placement may be smaller in caliber due to the altered spatial relationship between vessels, and therefore increases the difficulty for stent implantation.

Awareness of PV variations is also crucial for accurate tumor localization as for the branching pattern of PVs and hepatic veins determines the segmental anatomy of liver.

## Conclusion

In conclusion, variant PV anatomies are more commonly observed based on 3DVE than in previous reports using MDCT imaging alone. By providing a more comprehensive and intuitive description of liver segments and PV configurations, with better visualization than 2DCT, the 3DVT can demonstrate these variants in detail and is particularly important in accurate discrimination of the anatomy, especially among variations of the third-level branches (Type IV), parts of which have not been previously reported as PV subtypes. Awareness of PV variations is critically significant in performing safe and efficacious liver surgeries, such as portal vein embolization, liver resection, liver transplantation and TIPS creation. Knowledge of variant PV subtypes enables detection of PV variants during routine CT examination and can reduce iatrogenic morbidity and mortality.

## Data Availability

No datasets were generated or analysed during the current study.
